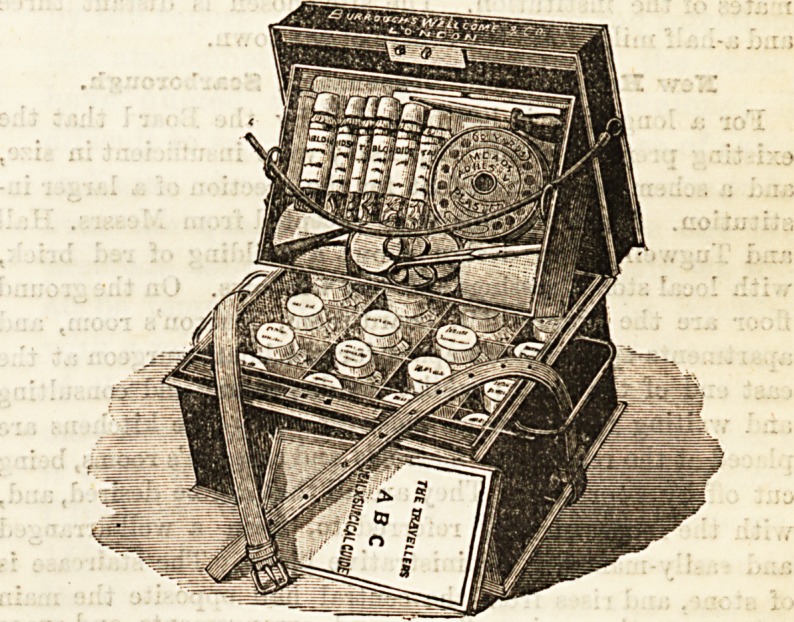# Emergency Case

**Published:** 1893-08-26

**Authors:** 


### EMERGENCY CASE.

Every traveller who is likely to be out of reach of doctors and chemists should provide himself with this most useful adjunct to his baggage. It is most convenient in size—some twelve inches in length by nine in height—made of black japanned tin provided with a small strap, which renders it quite easily carried by hand. Inside are to be found most of the little necessaries which should be at hand in any sudden advent of illness or accident. Generous supplies of oil silk, adhesive and court plasters, and mustard plasters, a good-sized packet of lint, a couple of calico bandages, Sacklin's linseed poultices, needles, lancet, stick of caustic, pair of scissors. All these are contained in the small tray at the top, together with a bottle of Hazeline cream. The lower division is in 16 compartments, each holding a very fair-sized bottle. These remedies are all in the form of tabloids, quinine being most largely represented in quantity. The little case is as compact as could be wished, and seems to contain all that is most likely to be needed. It will prove invaluable to wanderers in foreign lands, and can be obtained from Messrs. Burroughs, Wellcome, and Co., Snow Hill Buildings, London.

## Figures and Tables

**Figure f1:**